# Human Leukocyte Antigen (HLA) Influence on Prognosis of Autoimmune Hearing Loss

**DOI:** 10.3390/audiolres11010004

**Published:** 2021-01-25

**Authors:** George Psillas, Paris Binos, Grigorios G Dimas, Michalis Daniilidis, Jiannis Constantinidis

**Affiliations:** 1 1st Academic ENT Department, Aristotle University of Thessaloniki, AHEPA Hospital, 546 36 Thessaloniki, Greece; ikonstaa@auth.gr; 2 Department of Rehabilitation, Cyprus University of Technology, Archiepiskopou Kyprianou 30, Limassol 3036, Cyprus; paris.binos@cut.ac.cy; 3 1st Propedeutic Department of Internal Medicine, Aristotle University of Thessaloniki, AHEPA Hospital, 546 36 Thessaloniki, Greece; gregorydimas@yahoo.gr; 4 1st Internal Medicine Department, Aristotle University of Thessaloniki, AHEPA Hospital, 546 36 Thessaloniki, Greece; mdaniilidis@gmail.com

**Keywords:** hearing loss, autoimmunity, autoantibodies, HLA antigens, HLA-DRB1* alleles

## Abstract

Background: To evaluate the effect of human leukocyte antigen (HLA) on hearing outcome in patients suffering from autoimmune hearing loss (AIHL). Materials and Methods: The diagnosis of AIHL was essentially based on clinical symptoms, such as recurrent, sudden, fluctuating, or quickly progressing (<12 months) sensorineural hearing loss (uni-/bilateral). The molecular typing of HLA alleles was achieved by using polymerase chain reaction procedures. Patients underwent a tapering schema of steroid treatment and audiometric features were recorded. A logistic regression model was used to identify which HLA typing alleles were statistically significant in patients’ response to treatment. Results: Forty patients with AIHL were found to be carriers of HLA B27, B35, B51, C4, C7, and DRB1*04 alleles. No statistically significant influence of HLA B27, B35, B51, C4, C7, DRB1*04 HLA alleles typing was detected for the prognosis of AIHL. In these patients, the onset of AIHL was mainly progressive (53.8%), 29.2% of them had moderate hearing loss, and most of the cases had both bilateral hearing loss (62.5%) and downsloping audiogram (40%). Conclusion: The presence of HLA B27, B35, B51, C4, C7, and DRB1*04 alleles had no significant effect on a favorable outcome of AIHL. However, larger samples of patients are necessary in order to improve the knowledge about the HLA influence on the clinical course of AIHL.

## 1. Introduction

Autoimmune hearing loss (AIHL) is a specific clinical entity and may be related to the reaction antigen—autoantibody, the activation of the complement system, a direct action of cytotoxic T cells, or immune complex-mediated damage [1]. After the activation of the immune response and the release of interleukin (IL)-1β, immune-competent lymphocytes, and immunoglobulins can cause an excessive response involving cytokines, tumor necrosis factor, and interferon-gamma into the inner ear [2]. The result is the deposition of immune complexes into the endothelial surfaces of the labyrinthine vessels, microthrombosis, vascular changes and electrochemical alterations leading to damage to hair cells and spiral ganglion of the cochlea [3].

AIHL can present alone or is associated with other autoimmune systemic diseases (secondary AIHL), such as Cogan syndrome, Wegener granulomatosis, systemic lupus erythematosus, rheumatoid arthritis, Sjögren’s syndrome, ankylosing spondylitis, Susac syndrome, polyarteritis nodosa, Behcet’s disease, or Hashimoto’s thyroiditis [4]. Since McCabe [5] suggested autoimmune pathogenesis for sensorineural hearing loss, AIHL has always been recognized as a bilateral and rapidly progressive autoimmune disorder. In addition, other forms of HL have been identified to be immune-mediated in origin, such as (i) unilateral cases, in which serological nonspecific inner ear autoantibodies were found to be highly positive [6]; (ii) fluctuating hearing, which makes differential diagnosis between Meniere’s disease and AIHL difficult [7,8]; and (iii) unilateral [9] or bilateral [10,11] sudden sensorineural hearing loss, with or without relapses [12,13].

The major histocompatibility complex and human leukocyte antigen (HLA) associations have been rarely investigated for AIHL cases. The HLA system has been considered to be a disease marker for autoimmune diseases. HLA types such as HLA B27 [14], B35 [3,15], B51 [16], C4 [3,15], C7 [3,15] and DRB1*04 [17] have been considered as main markers for autoimmune inner ear diseases.

In this study, a possible effect of the HLA system on AIHL was assessed in order to better clarify the immunological characteristics of this inner ear disease.

## 2. Materials Methods

A retrospective study of patients with AIHL who were treated in the last 5 years was performed in a large tertiary referral center. The diagnosis of AIHL was essentially based on clinical symptoms, such as recurrent, sudden, fluctuating, or quickly progressing (<12 months) sensorineural hearing loss (uni-/bilateral). Hearing loss was considered progressive when it lasted several months and less than a year.

This study recruited 53 patients with AIHL, including ten males (age range: 20–60 year, mean: 36.2 ± 14.5 year) and 43 females (age range: 14–65 year, mean: 40.1 ± 14 year). Molecular typing of HLA class B27, B35, B51, C4, C7, and DRB1*01-32 was achieved by using polymerase chain reaction amplification with sequence-specific primers method (PCR-SSP) by a commercial kit (PEL FREEZ, Rogers, AR, USA). Molecular typing for class HLA -DP, -DQ, -DM, and -DN was not effectuated, due to unavailable material. The control group consisted of 371 healthy volunteers with no history of hearing loss, any ear disease, or autoimmune disease (180 males and 191 females, mean 42.5 ± 9 year), which were divided into 2 groups according to the HLA type tested [18,19]. The presence of concomitant systemic autoimmune diseases and specific laboratory markers, such as high levels of antinuclear autoantibodies (ANA titer values > 1:80), was also recorded. Patients with acoustic trauma, ototoxic drug exposure, Meniere’s disease, or retrocochlear lesion were excluded from our study.

A complete clinical history was obtained for all the patients, who were asked if they were also suffering from tinnitus and/or vestibular symptoms. Otoscopy and tympanogram studies were effectuated to exclude abnormal cases. Magnetic resonance imaging of the cerebellopontine angle and internal auditory canal was performed to exclude retrocochlear pathology, inner ear dysplasia, or enlarged vestibular aqueduct.

The initial pretreatment audiogram was assigned to one of the following five categories: (i) downsloping (left to right), (ii) upsloping (left to right), (iii) flat, (iv) cookie-bite (midfrequency hearing loss), and (v) inverse cookie-bite (low- and high-frequency hearing loss). The initial audiogram was classified into four categories, depending on the degree of hearing loss and based on the average of 6 frequencies (250, 500, 1000, 2000, 4000, 8000 Hz): (i) normal range (0 to 20 dB), (ii) mild (20 to 40 dB), (iii) moderate (40 to 60 dB), (iv) severe (60 to 80 dB), (v) profound (80 to 100 dB) and (vi) cophosis (over 100 dB).

Oral methylprednisolone was usually started at a dose of 48 mg per day (0.7 mg/kg/day) for four weeks. In the presence of improvement in hearing, the steroids were continued and gradually tapered over six months; if no response was noted after four weeks, tapering was scheduled for a maximum of 10 days [20]. A repeat course of steroids with a similar dose was applied in patients with fluctuations in hearing or recurrent presentations. Favorable responses to therapy were considered when there was a hearing improvement of 10 dB or more in at least three frequencies between 250 and 8000 Hz. Follow-up of all cases at least six months was required. At this time, the presence of different HLA typing was statistically related to the hearing outcome (favorable or not response) following steroid treatment. Clinical characteristics of AIHL were also recorded, including clinical onset and course, degree of hearing loss, and tonal audiogram configurations, in order to have a thorough aspect of this clinical entity.

### Statistical Analyses

The Mann–Whitney non-parametric test was used for two-group comparisons in continuous variables. Results are expressed as intermediate or interquartile range. Comparisons between categorical variables were evaluated by the X2 test. A logistic regression model was developed to identify which HLA typing alleles are statistically significant in patients’ response to treatment. For each variable, odds ratios (OR) and 95% confidence interval (confidence interval 95%) were calculated. All *p* values were two-sided, and a *p* of <0.05 was considered statistically significant. SPSS v.25.0 software package was used for all statistical analyses.

## 3. Results

Forty (75.4%) out of 53 patients suffering from AIHL were found to be carriers of HLA B27, B35, B51, C4, C7, and DRB1*04 alleles; however, the HLA B27 type was positive in only 2 patients. The remaining HLA DRB1 subtypes were identified in a limited number of patients to be evaluated. The frequency of HLA B27, B35, B51, C4, and C7 was lower compared to the control group (Table 1), except for HLA DRB1*04, which was slightly higher than 10% of the healthy control group. The HLA C04 typing was found to be the more frequent antigen in patients with AIHL. The majority of patients were females with associated autoimmune diseases in 37.5% of cases, predominantly with Hashimoto’s thyroiditis (Table 2).

Apart from hearing loss, most patients complained of tinnitus (75%) and less than half of them of vestibular symptoms (37.5%). The onset of AIHL was found to be mainly progressive (53.8%), 29.2% of cases had moderate HL, and most of the cases had both bilateral hearing loss (62.5%) and downsloping audiogram (40%) (Table 3).

During the follow-up, thirteen (13/40, 32.5%) patients showed a favorable response to steroid therapy. The post-treatment average hearing thresholds were better compared to the pre-treatment hearing thresholds for 250 and 4000 Hz [250 Hz, 30 ± 29 dB vs. 34 ± 24 dB; 4000 Hz, 46 ± 26 dB vs. 47 ± 23 dB], the same for 500 Hz [39 ± 28 dB vs. 39 ± 23 dB] and worse for 1000, 2000 and 8000 Hz [1000 Hz, 48 ± 26 dB vs. 43 ± 21 dB; 2000 Hz, 50 ± 23 dB vs. 48 ± 17 dB; 8000 Hz, 56 ± 33 dB vs. 54 ± 28 dB]. In the logistic regression model, non-carriers of HLA B27, B35, B51, and C07 were more likely to have a better response to steroid treatment than the carriers of HLA B27, B35, B51, and C07, but this was not statistically significant (*p* > 0.05). It was also more likely for the HLA C04 (OR > 1) and DRB1 04 carriers (OR > 1) to have a better response to steroid therapy, but this was equally not statistically significant (*p* > 0.05). Hence, the presence of the above HLA types did not statistically influence the prognosis of AIHL However, a trend for HLA C4 carriers (OR: 3.848) and HLA DRB1 04 (OR: 2.609) to have relatively better hearing levels following steroid treatment was noted, but this was not statistically significant (*p* > 0.05).

## 4. Discussion

The major histocompatibility complex and HLA associations have rarely been investigated for AIHL cases. The HLA system has been considered to be a disease marker for autoimmune diseases. The primary function of HLA molecules is the participation in antigen presentation, leading to T cell activation and B cell antibody production to clear infectious agents and malignant self-tissue and prevent autoimmunity by negative selection of autoreactive T cells. 

The role of the HLA class I (cluster of genes A, B, C) and class II (cluster of genes DR, DQ, DP) alleles as genetic factors has been studied in inner ear diseases. More specifically, regarding sudden hearing loss, it was supported that the presence of HLA-DQA1*03, -DQA1*05, and -DRB1*14 was associated with a poor recovery, and the presence of HLA-DQA1*01 and -DQB1*06 with good prognosis in Korean patients [21]. Cao et al. [22] studied 34 Belgian patients with idiopathic progressive sensorineural hearing loss and they observed an increased frequency of HLA DRB1*0301; they also found an increased incidence of DRB3*0101, DQB1*0201, and DPB1*0401. In another study [23], five of eight (62.5%) patients with progressive sensorineural hearing loss were DRB1*07 (DRB1*0701) for those typed at high resolution, compared with a normal American Caucasian frequency of 25.5%. Similarly, Bowman and Nelson [15] demonstrated a significant increase of C7 in 39 patients suffering from rapidly progressive sensorineural hearing loss. According to many studies, a high prevalence of Meniere’s disease was detected in carriers of the HLA-C7, C*0602, DRB1*1602, -A2, -B44, and -B27 alleles [24]. Melchiorri et al. [25] found a strong increase in the frequency of the HLA-C7 alleles (63.4%) in a cohort of 41 patients with Meniere’s disease, compared with healthy controls (35.6%) or with patients affected by other inner ear diseases (32.3%). However, a group from Spain [26] reported no association between 54 patients with definite Meniere’s disease and HLA-A, -B, -C, or -DR alleles compared with normal controls. In general, the HLA markers are dependent on the ethnic origin, and the statistical correlations with the hearing outcome could be different in each population.

In our study, no statistically significant influence of B27, B35, B51, C4, C7, DRB1*04 HLA alleles typing was detected for the prognosis of AIHL; nevertheless, the HLA DRB1*04 alleles were found to be slightly higher compared with the healthy control group (Table 1). The HLA–DRB1*04 alleles have been reported to be a risk factor for presumed autoimmune diseases that also affect the inner ear, such as Vogt–Koyanagi–Harada disease [27]; likewise, the presence of HLA–DRB1*04 subtypes, including DRB1*0401, DRB1*0404, DRB1*0405 and DRB1*0408 were associated with high risk for developing rheumatoid arthritis [28]. As the inner ear diseases is concerned, the HLA DRB1*04 has been reported to have a prognostic value in patients suffering from sudden sensorineural hearing loss [17,21]; those patients who carried the HLA DRB1*04 alleles had a poor outcome. Hence, the frequency of HLA DRB1*04 was increased in patients who did not respond to steroid therapy compared with patients who responded well to steroid therapy. In contrast, our study showed that the HLA DRB1*04 carriers have a possibly more favorable recovery, but this result was without statistical significance. Moreover, in our study, the HLA C04 typing was found to be the more frequent antigen in patients with AIHL. Our analysis also showed that the HLA C04 typing may have a better prognosis, but this effect on AIHL was not statistically significant (Table 4).

Among the nonspecific autoimmune testings, ANA was relatively elevated in our sample (35%) (Table 2). In general, the sensitivity of the ANA test for autoimmune diseases is very high, but the specificity is quite low; the ANA result must be interpreted in the specific context of every individual patient’s symptoms [2,7]. In our study, specific autoimmune testings, such as the lymphocyte migration inhibition assays, the lymphocyte transformation test, and assessments of tumor necrosis factor and anti-cochlear antibodies have not been used, as their diagnostic accuracy for AIHL remains undetermined [20]. For example, an anti-68-kDa autoantibody in the sera of patients with rapidly progressive sensorineural hearing HL was identified by Western blotting (HSP-70) [29]; however, in AIHL patients, the frequency of antibodies against HSP-70 did not differ between patients and controls and was not useful in the diagnosis of AIHL [30]. In addition, Hashimoto’s thyroiditis was the most prevalent coexisted autoimmune disease (27.5%, Table 2). Arduc et al. [31] indicated that Hashimoto’s thyroiditis could affect the auditory organ, and hearing involvement should be considered one element in the clinical picture of this disease.

Steroid therapy remains the mainstay of treatment for AIHL due to its immunosuppressive properties. However, in our study, approximately one third (32.5%) of patients responded favorably to steroid therapy. Many studies [7,10,11,32,33,34] found that the effect of steroids on AIHL was not so beneficial, only 43% to 70% of patients with AIHL responded successfully to steroids, and over time, a further deterioration of hearing was noted. The real efficacy of steroid treatment was estimated to be only 14% [2], as indicated by our hearing outcome results.

Larger samples of patients are necessary in order to improve the knowledge about the HLA influence on the clinical course of AIHL. A better understanding of the association of HLA molecules with autoimmune diseases is needed, since the underlying pathogenetic mechanisms have not yet been clarified. The assessment of more targeting HLA alleles and subtypes is expected in our future studies to thoroughly evaluate the relationship between AIHL and autoimmune pathways. Treatment of steroid-resistant AIHL is also a challenge and multicentric trials and collaboration are required to achieve more effective therapeutic interventions.

## Figures and Tables

**Table 1:** Frequency of HLA B27, B35, B51, C4, C7 and DRB1*04 in group of patients and in control groups.

	Patients (%) (2n Alleles) (*n* = 53)	Healthy Control (%) (*n* = 246) [18]
HLA B27	2 (1.8)	2
HLA B35	15 (14.1)	14.5
HLA B51	9 (8.4)	15.1
HLA C4	17 (16)	16.5
HLA C7	15 (14.1)	17.7
	**Patients (%) (2n Alleles)** **(*n* = 53)**	**Healthy Control (%)** **(*n* = 125) [19]**
HLA DRB1*04	11 (10.3)	10

**Table 2:** Demographic data, laboratory findings (ANA) and coexisted autoimmune diseases in patients with autoimmune hearing loss and carriers of HLA B27, B35, B51, C4, C7, and DRB1*04 alleles (*n* = 40 cases).

Age (average age ± SD, in years)	38.3 ± 14.6
Gender (M/F)	8/32
Antinuclear autoantibodies (ANA)	14 (35%)
Other autoimmune disease	15 (37.5%)
- Hashimoto’s thyroiditis	11 (27.5%)
- Rheumatoid arthritis	2 (5%)
- Lupus erythematosus	1 (2.5%)
- Idiopathic thrombocytopenic purpura	1 (2.5%)

**Table 3:** Audiological symptoms and initial audiometric characteristics in patients (40 cases, 65 ears) with autoimmune hearing loss and carriers of HLA B27, B35, B51, C4, C7, and DRB1*04 alleles.

Affected side (R/L)	29 ears /36 ears
Unilateral/bilateral hearing loss	15 cases (37.5%)/25 cases (62.5%)
Presence of tinnitus	30 cases (75%)
Presence of vestibular complaints	15 cases (37.5%)
Onset of hearing loss	
- sudden	24 ears (36.9%)
- fluctuating	6 ears (9.2%)
- progressive	35 ears (53.8%)
Recurrence	
- yes	16 ears (24.6%)
- no	49 ears (75.3%)
Slope of tonal audiogram	
- downsloping	26 ears (40%)
- upsloping	17 ears (26.1%)
- flat	14 ears (21.5%)
- cookie-bite	7 ears (10.7%)
- inverse cookie-bite	1 ear (1.5%)
Degree of initial hearing loss	
- mild	16 cases (24.6%)
- moderate	19 cases (29.2%)
- severe	4 cases (6.1%)
- profound	1 case (1.5%)

**Table 4:** Logistic regression analysis of HLA alleles of patients suffering from autoimmune hearing loss related to their response to treatment. yes: favorable response to therapy, no: no response to therapy.

	B Value	*p*-Value	Exp(B) = Odds Ratio	Lower Limit (95% C.I. for Exp B)	Upper Limit (95% C.I. for Exp B)
HLA B27 (yes/no)	−1.049	0.608	0.350	0.006	19.231
HLA B35 (yes/no)	−2.539	0.155	0.079	0.002	2.619
HLA B51 (yes/no)	−0.106	0.942	0.900	0.052	15.536
HLA C04 (yes/no)	1.348	0.396	3.848	0.171	86.363
HLA C07 (yes/no)	−2.204	0.099	0.110	0.008	1.512
HLA DRB1*04 (yes/no)	0.959	0.395	2.609	0.287	23.735

## Data Availability

Data available on request due to privacy.
